# Males and Females Have Distinct Molecular Events in the Articular Cartilage during Knee Osteoarthritis

**DOI:** 10.3390/ijms22157876

**Published:** 2021-07-23

**Authors:** Chenshuang Li, Zhong Zheng

**Affiliations:** 1 Department of Orthodontics, School of Dental Medicine, University of Pennsylvania, Philadelphia, PA 19104, USA; lichens@upenn.edu; 2 Division of Growth and Development, Section of Orthodontics, School of Dentistry, University of California, Los Angeles, CA 90095, USA; 3 Department of Surgery, David Geffen School of Medicine, University of California, Los Angeles, CA 90095, USA

**Keywords:** sex as a biological variable, osteoarthritis, cartilage, whole transcriptome sequencing, molecules

## Abstract

Osteoarthritis (OA) is a major public health challenge that imposes a remarkable burden on the affected individuals and the healthcare system. Based on the clinical observation, males and females have different prevalence rates and severity levels of OA. Thus, sex-based differences may play essential roles in OA’s prognosis and treatment outcomes. To date, the comprehensive understanding of the relationship between sex and OA is still largely lacking. In the current study, we analyzed a published transcriptome dataset of knee articular cartilage (GSE114007) from 18 healthy (five females, 13 males) and 20 OA (11 females, nine males) donors to provide a slight insight into this important but complex issue. First, comparing female healthy cartilage samples with those of males revealed 36 differential expression genes (DEGs), indicating the fundamental sex-related differences at the molecular level. Meanwhile, 923 DEGs were distinguished between OA and healthy female cartilage, which can be enriched to 15 Reactome pathways. On the other hand, when comparing OA and healthy male cartilage, there are only 419 DEGs were identified, and only six pathways were enriched against the Reactome database. The different signaling response to OA in the male and female cartilage was further enforced by recognizing 50 genes with significantly different OA-responsive expression fold changes in males and females. Particularly, 14 Reactome pathways, such as “Extracellular matrix organization”, “Collagen biosynthesis and modifying enzymes”, “Dissolution of fibrin clot”, and “Platelet Aggregation (Plug formation)”, can be noted from these 50 sex-dependent OA-responsive genes. Overall, the current study explores the Sex as a Biological Variable (SABV) at the transcriptomic level in the knee articular cartilage in both healthy status and OA event, which could help predict the differential OA prognosis and treatment outcome of males and female patients.

## 1. Introduction

As the most common form of arthritis, osteoarthritis (OA) is a series of pathology that causes persistent pain, swelling, and reduced motion in the affected joints. For years, OA was identified as an age-related pathology; thus, it has been called “wear and tear” arthritis. During the past few years, OA is increasingly recognized as a highly heterogeneous group of diseases characterized by variable clinical phenotypes, which may contribute to the inconsistency of clinical prognosis and treatment response [1].

With the growing recognition of Sex as a Biological Variable (SABV) in the pathophysiology of a diversity of diseases [2], the impact the sex on OA has also attracted more and more attention. To date, it is well known that OA has a higher prevalence in women than men, as 62% of OA patients are women [3]. Indeed, women have a consistently higher OA prevalence rate than men in all age groups between the 30s to 95 plus [4]. Worldwide estimates are that 9.6% of men and 18% of women aged over 60 have symptomatic OA [5]. Moreover, disability and loss of function associated with OA are higher in women [6,7]. Besides, the US Medical Expenditure Panel Survey data for the years 1996 to 2005 found that OA-related out-of-pocket (OOP) costs incurred by women were greater than those by men [8], and more women than men were hospitalized for OA [9]. In the US, OA increased annual per capita absenteeism costs of $5.5 billion for female workers verse $4.8 billion for male workers [8].

Clinically, the incidence of OA increases dramatically in women around the time of menopause [10]; therefore, the modulating role of sex hormones on OA was proposed [11]. For example, estrogen is one of the most deeply investigated sex hormones in OA [12]. Although estrogen is considered to have protective potency against OA, the effects of estrogen replacement therapy and selective estrogen receptor modulators in preserving and/or restoring joint tissue in OA are controversial among currently published reports [13,14]. Besides estrogen, sex hormone-binding globulin [15], follicle-stimulating hormone [16], dehydroepiandrosterone [17], progesterone [18], and testosterone [19] may all influence OA progression. However, none of these sex hormones can completely explain all differences observed between male and female OA patients [20]. For instance, at a macro level, males and females have different thicknesses of cartilage [21], subchondral bone density [22], and muscle strength [23]; while at a micro level, tissue and cells from females have different, or even distinct, responses in comparison with those from males [20]. Recently, Kim et al. [24] found that OA-related studies were largely performed in male subjects and animals, although females face more OA risk and more server symptoms [4,5,6,7]. Undoubtedly, fully considering SABV will set the fundamental to understanding the distinguished clinical complaints between males and females and is an essential step for effective therapy development, which, unfortunately, is still largely lacking.

Although synovium and subchondral bone are known to involve in OA recently, articular cartilage is still the major target of OA-related investigations. Articular cartilage is hyaline cartilage that does not have blood vessels, nerves, or lymphatics [25]. It is composed of a dense extracellular matrix (ECM) with a sparse distribution of chondrocytes. The major components of the ECM are water, collagen, and proteoglycans, which are critical to maintaining the mechanical property of the cartilage [25]. In a healthy microenvironment, the balance between cartilage synthesis and degradation is strictly regulated [26]. In the OA scenario, chondrocytes express more catabolic molecules, such as matrix metallopeptidase 13 (MMP-13), and less anabolic matrix, such as type II collagen [27,28], and thus matrix remodeling, inappropriate hypertrophy-like maturation, and cartilage calcification appear [29]. A net loss of proteoglycan content is also one of the hallmarks of all stages of OA cartilage degeneration [26]. In addition to the well-known anabolic and catabolic components, increasingly more biological factors have been noted to participate in OA’s molecular events. For instance, nerve growth factor (NGF), which was primarily discovered for its roles in sensory neuron proliferation and sensitization, is recently reported to regulate articular chondrocytes’ calcification [30]. Another example is C1q and TNF related 1 (C1QTNF1), whose modulating effects on chondrocyte proliferation and maturation is revealed recently, belongs to a newly discovered family of highly conserved adiponectin paralog proteins [31]. Therefore, a more detailed dissection of the molecular events in the OA cartilage is needed to assist the understanding of SABV in OA pathophysiology.

## 2. Results

### 2.1. Male and Female Cartilage Are Not Molecularly Identical in the Healthy Status

We first compare the mRNA sequencing data from the male and female healthy cartilage to investigate if the transcriptomic profiles are the same for both genders. Within the 23,714 identified genes, the expression of the commonly used cartilage anabolic markers, such as *Collagen Type II Alpha 1 Chain* (*COL2A1)*, *Aggrecan* (*ACAN*), *cartilage oligomeric matrix protein* (*COMP*), and *SRY-box 9* (*SOX9*), and catabolic markers, such as *Runt-related transcription factor2* (*Runx2*), *MMP13*, *ADAM metallopeptidase with thrombospondin type 1 motif 4* (*ADAMTS4*), and *ADAMTS5*, are not significantly different between the healthy male and female cartilage (Supplemental Table S1).

**Figure 1:**
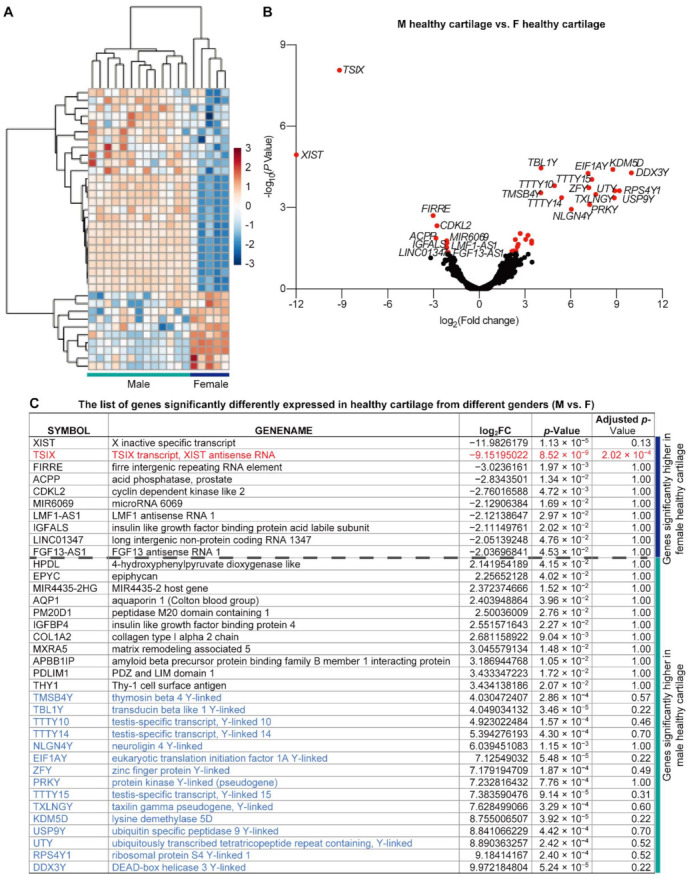
The differential expressed genes (DEGs) detected between male and female healthy knee cartilage samples. (**A**) Heatmap and (**B**) volcano diagrams for DEG visualization. DEGs with a *p*-value less than 0.05 are highlighted in red. (**C**) The list of genes that are significantly differentially expressed in healthy male and female cartilage. DEGs with a statistically significant higher level in females have a negative log_2_FC value, while those highly expressed in males have a positive log_2_FC value. The gene with an adjusted *p*-value less than 0.05 is highlighted in red. The Y-chromosome linked genes are highlighted in blue font.

On the other hand, we identify 10 DEGs with a *p*-value less than 0.05 that are highly expressed in healthy female cartilage than their male counterparts, and 26 DEGs with a *p*-value less than 0.05 whose expression level is lower in females (Figure 1). For all these 36 DEGs, only *TSIX transcript, XIST antisense RNA* (*TSIX*) has an adjusted *p*-value less than 0.05 (Figure 1C and Supplemental Table S1, highlighted in red). Among the latter 26 genes whose expression levels are lower in females, 15 genes are Y-chromosome linked (Figure 1C), demonstrating the reliability of the current study. Thus, the different expression levels of non-Y-chromosome-linked genes between males and females may present the SABV at a molecular level (Figure 1).

Pathway enrichment was used to uncover the potential functional interaction among these 36 DEGs, while only 14 genes could be recognized by the Reactome knowledgebase. DEGs that were not recognized by the current Reactome database are summarized in Supplemental Table S2. The Reactome recognized genes were clustered into “chromatin organization”, “hemostasis”, “disease”, “metabolism”, “transport of small molecules”, “metabolism of proteins”, and “extracellular matrix organization.” Among them, nine identified pathways have a *p*-value less than 0.05, but none of them qualified as a significant enrichment that should have an FDR smaller than 0.05 (Table 1 and Supplemental Table S3).

### 2.2. ECM Organization Is the Major Event in OA Cartilage of Females, But Not That of Males

**Figure 2:**
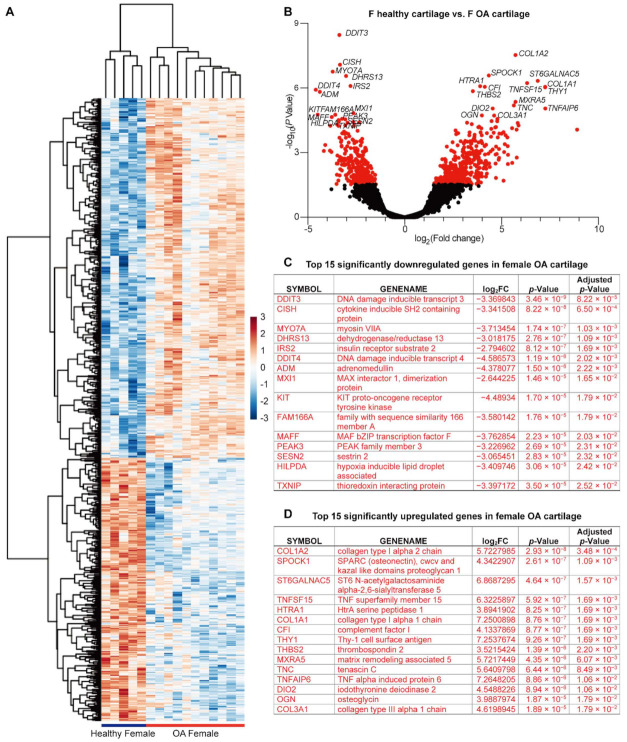
The DEGs detected between female healthy and OA cartilage. (**A**) Heatmap and (**B**) volcano diagrams for DEG visualization. DEGs with a *p*-value less than 0.05 are highlighted in red. (**C**) Top 15 genes significantly downregulated in female cartilage in response to OA. (**D**) Top 15 genes significantly upregulated in female cartilage in response to OA. DEGs with an adjusted *p*-value less than 0.05 are highlighted in red.

We then analyzed the cartilage gene expression changes during OA of males and females separately. First, in the female cartilage, there were 923 DEGs in total, among which 382 were downregulated and 541 were upregulated during OA (Figure 2 and Supplemental Table S4). Among these genes, 30 significantly downregulated DEGs and 45 upregulated ones were identified with an adjusted *p*-value less than 0.05 (Supplemental Table S4, highlighted in red). Ranking based on the *p*-values, the top 15 significantly downregulated genes were summarized in Figure 2C, while the top 15 significantly upregulated in Figure 2D.

In the Reactome knowledgebase, 424 of the 923 DEGs could not be matched (Supplemental Table S5); thus, the pathways were enriched based on the other 499 DEGs. Overall, there were 68 pathways with a *p* < 0.05, among which 15 pathways with an FDR less than 0.05 (Table 2 and Supplemental Table S6). Nine of the 15 pathways are related to ECM organization (Table 2). For the other six pathways, “FOXO-mediated transcription of cell cycle genes”, “FOXO-mediated transcription”, and “RUNX3 regulated immune response and cell migration” belong to the event “gene expression (transcription)”, “Response of EIF2AK1 (HRI) to heme deficiency” belongs to the event “cellular responses to external stimuli”, “Interleukin-4 and Interleukin-13 signaling” belongs to the event “immune system”, and “Gap junction assembly” belongs to the event “vesicle-mediated transport” (Table 2). In particular, 55 of 499 identified DEGs were enriched in “extracellular matrix organization”, which is the most significant event in the female cartilage in response to OA.

**Figure 3:**
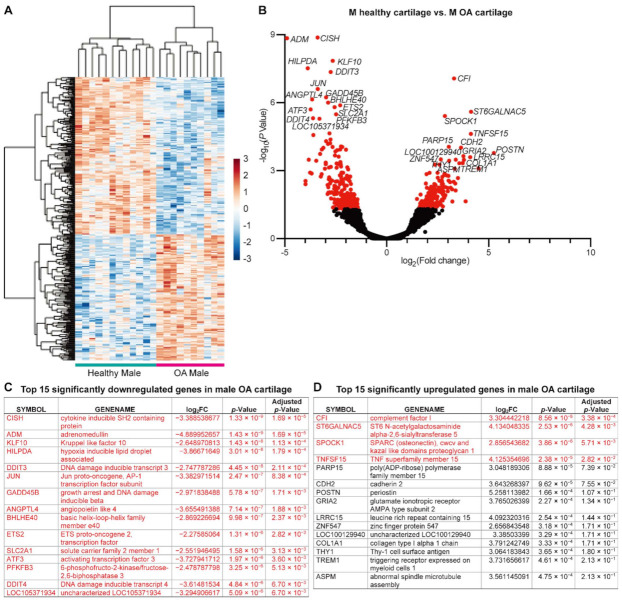
The DEGs detected between male healthy and OA cartilage. (**A**) Heatmap and (**B**) volcano diagrams for DEG visualization. DEGs with a *p*-value less than 0.05 are highlighted in red. (**C**) Top 15 genes significantly downregulated in male cartilage in response to OA. (**D**) Top 15 genes significantly upregulated in male cartilage in response to OA. DEGs with an adjusted *p*-value less than 0.05 are highlighted in red.

Second, we analyzed the male cartilage in the same way. Male samples have much less OA-responsive DEGs compared with female samples. There were 419 DEGs in total, 186 upregulated and 233 downregulated, among which 18 downregulated and four upregulated DEGs have an adjusted *p*-value less than 0.05 (Figure 3 and Supplemental Table S7, highlighted in red). In addition, the top 15 significant upregulated and downregulated genes based on *p*-value in male cartilage during OA were not as same as those in female cartilage. The top 15 significantly downregulated genes in male cartilage were listed in Figure 3C, while the top 15 significantly upregulated genes in Figure 3D.

In the Reactome knowledgebase, 202 of the 419 DEGs could not be matched (Supplemental Table S8). Thus, the pathways enrichment based on the other 217 DEGs dispersed the molecular events including “immune system”, “signal transduction”, “neuronal system”, “hemostasis”, “gene expression (transcription)”, “metabolism”, “DNA replication”, “transport of small molecules”, “disease”, “metabolism of proteins”, “cell cycle”, “autophagy”, “vesicle-mediated transport”, “cellular responses to external stimuli”, and “extracellular matrix organization”. There are 79 pathways that have a *p*-value less than 0.05, among which six have an FDR less than 0.05 (Table 3 and Supplemental Table S9). Here, “Response of EIF2AK1 (HRI) to heme deficiency” belongs to the event “cellular responses to external stimuli”, “ATF4 activates genes in response to endoplasmic reticulum stress” and “PERK regulates gene expression” belong to the event “metabolism of proteins”, “NGF-stimulated transcription” and “Nuclear Events (kinase and transcription factor activation)” belong to the event “signal transduction”, and “MECP2 regulates neuronal receptors and channels” belongs to the event “gene expression (transcription)”. None of these six pathways are categorized in the event of “extracellular matrix organization”.

### 2.3. Male and Female Cartilage Have Significant Different Alteration Genes during OA

To confirm the differences between male and female cartilage in response to OA as observed above, we also compared the gene expression fold change in both sexes and identified 63 DEGs with a *p* < 0.05 (Supplemental Table S10). By referencing the single-sex OA—healthy cartilage comparison results, genes that do not have OA-responsive alteration(s) in either gender were excluded to eliminate the false positive result and lead to the identification of 50 DEGs (Table 4). Note that none of these genes were detected with an adjusted *p*-value less than 0.05, while 23 of these 50 DEGs could not be recognized by Reactome (Supplemental Table S11). Based on the 27 Reactome-recognized genes, 60 pathways were enriched (*p* < 0.05; Supplemental Table S12). Among them, 14 pathways have an FDR less than 0.05, which could be clustered in the events of “Extracellular matrix organization” (including “Extracellular matrix organization”, “Collagen biosynthesis and modifying enzymes”, “Collagen chain trimerization”, “Collagen formation”, “Assembly of collagen fibrils and other multimeric structures”, “Collagen degradation”, “ECM proteoglycans”, “Integrin cell surface interactions”, and “Anchoring fibril formation”), “Hemostasis” (including “Dissolution of Fibrin Clot”, “GP1b-IX-V activation signaling”, “Platelet Aggregation (Plug Formation)”, and “Platelet Adhesion to exposed collagen”), and “Disease” (including “Diseases of glycosylation”) (Table 5). These results further validate male and female cartilage differences at the molecular event level in response to OA.

## 3. Discussion

It is broadly accepted that exploring the OA-responsive biomarkers shared by both genders will pave the path for developing the therapeutics that benefit both male and female OA patients [32]. On the other hand, the distinguished clinical appearance between male and female patients warrants the mechanistic investigation at the molecule level. In the current study, the global gene expression profiles of knee joint articular cartilage from male and female donors of a well-accepted dataset [33,34,35,36,37,38,39,40,41,42], GSE114007, were comprehensively compared to gain insight into the understanding of the SABV not only in the healthy status, but also in the response of OA stimulation.

Firstly, the 36 identified male-vs.-female DEGs in healthy cartilage confirmed the hypothesis that the SABV is not limited to the thickness and articular surface areas [21,43] but extended to the static transcriptomic level. In particular, besides the 15 Y-chromosome-linked genes, several genes among the 36 male-vs.-female DEGs in healthy cartilage have been correlated with OA development and progression. For example, as an intensively investigated long non-coding RNA (lncRNA), *XIST* is highly expressed in OA cartilage tissue and IL-1β-treated chondrocytes [44] and has anti-apoptosis and chondroprotective effects [45]. On the other hand, another lncRNA, *MIR4435-2HG*, is downregulated in OA [46] and may have inhibition effects on the progression of OA [47]. Regarding the ECM components, a small leucine-rich proteoglycan (SLRP), epiphycan, plays an important role in maintaining joint integrity, and *epiphycan*-deficient mice spontaneously develop OA with age [48]; *Col1A2* is one of the typical markers for fibrocartilage [49] and *MXRA5* is highly expressed in the synovial fluid of OA patients [50]. Some other DEGs identified in our current studies have also been associated with OA in previous investigations. For instance, *PDLIM1* is downregulated in IL-1β-treated chondrocytes [51], *THY1* is highly expressed in OA cartilage and could be induced by IL-1β [52], and *EIF1AY* has been identified as one of the 9 OA diagnostic biomarkers [53]. In addition, AQP1 promotes caspase-3 activation and thereby contributes to chondrocyte apoptosis [54], and thus the activation of AQP1 induced by OA process can be used to control the tissue degeneration [55].

In addition, *IGFBP4* has been identified as the late response gene of parathyroid hormone-related protein (PTHrP) in chondrocytes [56]. It functions as an IGF-1 inhibitor and participates in the inflammatory response [57]. Meanwhile, *IGFALS* encodes a serum protein that binds IGFs to increase their half-life and vascular distribution [58]. As the male healthy articular cartilage has a lower expression level of *IGFALS* and higher expression level of *IGFBP4* than female cartilage, we infer that IGF-1 signaling is less activated in male cartilage than their female counterpart.

Note that among these 36 DEGs, only *TSIX* has an adjusted *p*-value less than 0.05, indicating the significance of TSIX for gender-dependent biological differences in the articular cartilage. However, the detailed function of TSIX in cartilage remains blank. In addition, the limited available sample could lead to only one DEG identified with an adjusted *p*-value less than 0.05 identified, while more DEGs with a *p*-value less than 0.05 (36 DEGs) were recognized. Thus, further studies are undoubtedly encouraged to fully understand the SABV in healthy knee articular cartilage at the molecular level, which warrants a worldwide collaboration for more database collection in a diverse of populations.

Interestingly, when we profile OA-responsive transcriptional changes in male and female cartilage separately, the amount of OA-responsive DEGs with an adjusted *p*-value less than 0.05 in female cartilage is triple that in male cartilage, indicating more intense OA-induced molecular changes in female cartilage than that in male counterparts. This transcriptomic difference could be correlated with the clinical observation that women experience more severe OA symptoms than men [59,60]. Considering the different total amounts of OA-responsive DEGs, it is no surprise to find that the top 15 OA-responsive upregulated and downregulated DEGs are not identical in male and female cartilage. In fact, male and female cartilage do share some top OA-responsive DEGs with an adjusted *p*-value less than 0.05, such as *CISH*, *ADM*, *HLPDA*, *DDIT3*, *DDIT4*, *CFI*, *ST6GALNAC5*, *SPOCK1*, and *TNFSF15*. Regarding the Reactome-enriched pathways, “response of EIF2AK1 (HRI) to heme deficiency” is the common significant pathway with adjusted *p*-value less than 0.05 in response to OA stimulation shared by male and female cartilage. These shared genes and pathways could be considered as potential targets for OA diagnosis and treatment, which can benefit both genders.

The OA-responsive molecular events in female cartilage are tightly clustered in the “extracellular matrix organization”, which could explain the reason that female patients have more severe OA-related cartilage defects than males [60,61]. Meanwhile, “FOXO-mediated transcription”, “RUNX3-regulated immune response and cell migration”, and “Interleukin-4 and Interleukin-13 signaling”, the pathways with FDR less than 0.05, might be additional key pathways to regulate OA in females. In fact, recent studies demonstrate that FOXO transcription factors modulate autophagy and proteoglycan 4 in cartilage, and conditional knockout FOXOs could induce OA-like changes in the mice [62,63]. On the other hand, ECM degradation does not present as the leading OA-responsive event in the male cartilage. Instead, “ATF4 activates genes in response to endoplasmic reticulum stress”, “NGF-stimulated transcription”, “MECP2 regulates neuronal receptors and channels”, “PERK regulates gene expression”, and “Nuclear Events (kinase and transcription factor activation)” were enriched from the OA-responsive DEGs in male cartilage with FDR less than 0.05, indicating a distinct molecular response to OA between male and female cartilage. The activation of the PERK-ATF4-CHOP axis is especially known to mediate impaired cartilage function [64]; however, the effects of these male-specific OA-responsive pathways in arthritis are still unknown.

SABV of cartilage in response to OA was further evaluated by comparing the OA-response DEGs from both genders directly, by which 50 genes with significantly different expression fold changes were identified, but none of the genes has an adjusted *p*-value less than 0.05. As expected, “Extracellular matrix organization” is the major sex-relative differential event harboring 9 of the 14 enriched pathways. There are also differences in “Hemostasis” and “Diseases of glycosylation” events. Note that several genes clustered in the event “Hemostasis” (including pathways “Dissolution of Fibrin Clot”, “GP1b-IX-V activation signaling”, “Platelet Aggregation (Plug Formation)”, and “Platelet Adhesion to exposed collagen”) have also been investigated in OA-related area. For example, *SERPINE1* has been identified as one of the OA-specific genes in human joint fibroblast-like synoviocytes [65]. While *SERPINE2*, a contributor for both “Hemostasis” and “Diseases of glycosylation” events, upregulated by IL-1α stimulation in human chondrocytes, and recombinant SERPINE2 may protect chondrocytes by inhibiting MMP-13 expression [66]. Besides, high platelet counts within the normal range are significantly associated with knee and hip OA in women aged above 50 [67].

Considering aging may be an important indicator of OA, it is not a surprise that the donors of the OA groups are older than the healthy group when the dataset was built [33]. Interestingly, specifically grouping the samples in the same dataset GSE114007 by donor age, Chen et al. concluded that age is not a dependent variable for differentially expressed gene identification [41]. Here, as demonstrated in Table 6, no difference regarding donor age between males and females was found in healthy cartilages nor OA samples. Thus, the age contribution on OA-responsive differentially expressed genes, if any, has already been considered in parallel for both genders. Note that comparing healthy cartilage of different age stages for each gender would be an interesting and important topic for gaining more insight on the molecular events in senescence, particularly in a gender-dependent manner. Besides, an inter-cohort validation should be conducted in the future to verify the genes and pathways discovered in the current study. Last but not least, it is the first time that multiple genes and pathways mentioned above are associated with chondrogenic differentiation, maintenance, and pathology. The underlying mechanistic and functional details are largely unknown. No doubt, a huge amount of effort should be devoted on a global base to transferring the discovery here to the real world. 

## 4. Materials and Methods

By using the keywords “osteoarthritis” and “cartilage” in the NCBI GEO DataSets website [68] with the selection of “*Homo sapiens*” under the column of “Top Organisms” and “Expression profiling by high throughput sequencing” under the column of “study type”, 31 series were identified. After reviewing all these datasets to check if they provided the sex information of the donors, one series (GSE114007) containing transcriptome data of human knee cartilage samples was included in the current study [33]. In this dataset, there were samples from 5 healthy female donors (age 27–57, mean 42), 13 healthy male donors (age 18–61, mean 34.5), 11 OA female donors (age 52–82, mean 66.3), and 9 OA male donors (age 51–71, mean 64.9) (Table 6 and Supplemental Table S13). According to the original study of this dataset, there is no significant difference between healthy and OA samples in other factors, such as the health condition of the donors, tissue sampling location, and body mass index [33,41]. SRA data of all the samples were downloaded from NCBI SRA website [69]. Following comparisons were conducted: (1) male healthy (HM) cartilage with female healthy (HF) cartilage to explore the baseline molecular differences in the articular cartilage between genders, (2) male OA (OM) cartilage with HM cartilage to detect the molecular changes in response to OA in males, (3) female OA (OF) cartilage with HF cartilage to detect the molecular changes in response to OA in females, and (4) OA-responsive DEGs in males (OM-HM) with that in females (OF-HF) to find the genes altered significantly different between genders during OA (OM-HM vs. OF-HF). Data analyses were performed on the Galaxy platform (UseGalaxy.org; [70]) with an established, broadly validated protocol [71,72,73]. Briefly, the FASTQC RNA-seq reads were aligned to the human genome (GRCh38) using HISAT2 aligner (Galaxy Version 2.1.0+galaxy 5) with default parameters [74]. Raw counts of sequencing read for the feature of genes were extracted by featureCounts (Galaxy Version 1.6.4+galaxy1) [75]. Then, the limma package (Galaxy version 3.38.3 + galaxy3) was used to identify DEGs with its *voom* method [76,77]. Expressed genes were selected as their counts per million (CPM), value not less than 1 in at least two samples across the entire experiment, while lowly expressed genes were removed for the flowing analyses. The parameters were set as 1 for minimum log_2_ Fold change and 0.05 for *p*-value adjustment threshold. As our current investigation is an explorative study, Benjamini–Hochberg correction was employed in the limma-voom analysis for *p* value adjustment [78], which is highly recommandated by the limma user guide [79]. To provide FDR control, the limma Test significance relative to a fold-change threshold (TREAT) function was applied to select genes that are more likely to be biologically significant [80], accompanied by the Robust Setting to protect against outlier genes [81]. A trimmed mean of M values (TMM) method was used for normalization among RNA samples. Quasi-likelihood F-tests (ANOVA-like analysis) were achieved to identify DEGs [82]. Genes with fold change (FC) more than 2 and *p* value less than 0.05 were assigned as DEGs. Heatmap diagrams were conducted in *R* (version 3.6.3) [83] with packages *pheatmap* (version 1.0.12), while volcano plots were generated by GraphPad Prism (version 8.2.1; GraphPad Software, Inc., San Diego, CA, USA). Pathway enrichment of identified DEGs was performed against the Reactome knowledgebase [84]. The enriched pathways with a false discovery rate (FDR) less than 0.05 were considered significantly meaningful.

## 5. Conclusions

In summary, our current study confirmed SABV in the knee cartilage at the transcriptomic level in both healthy and OA statuses. This study, at least partially, explains the clinical observed sex-relative differences of OA outcomes. Due to the lack of knowledge about some of the identified DEGs, further worldwide collaboration is necessary to comprehensively uncover the sex-relative differences of knee articular cartilage health and disease.

## Figures and Tables

**Table 1:** The pathway enrichment result of the significant male-vs.-female DEGs in healthy cartilage against the Reactome knowledgebase (*p* < 0.05). Note: no pathways have an FDR value less than 0.05.

Pathway Identifier	Pathway Name	#Entities Found	#Entities Total	Entities Ratio	Entities *p*-Value	Entities FDR	Submitted Entities Found
R-HSA-3214842	HDMs demethylate histones	2	31	2.11 × 10^−3^	2.96 × 10^−3^	2.46 × 10^−1^	KDM5D; UTY
R-HSA-76009	Platelet Aggregation (Plug Formation)	2	53	3.60 × 10^−3^	8.36 × 10^−3^	2.46 × 10^−1^	APBB1IP; COL1A2
R-HSA-9673163	Oleoyl-phe metabolism	1	5	3.40 × 10^−4^	1.28 × 10^−2^	2.46 × 10^−1^	PM20D1
R-HSA-430116	GP1b-IX-V activation signaling	1	12	8.15 × 10^−4^	3.05 × 10^−2^	2.46 × 10^−1^	COL1A2
R-HSA-2214320	Anchoring fibril formation	1	15	1.02 × 10^−3^	3.80 × 10^−2^	2.46 × 10^−1^	COL1A2
R-HSA-75892	Platelet Adhesion to exposed collagen	1	16	1.09 × 10^−3^	4.05 × 10^−2^	2.46 × 10^−1^	COL1A2
R-HSA-1247673	Erythrocytes take up oxygen and release carbon dioxide	1	16	1.09 × 10^−3^	4.05 × 10^−2^	2.46 × 10^−1^	AQP1
R-HSA-381426	Regulation of Insulin-like Growth Factor (IGF) transport and uptake by Insulin-like Growth Factor Binding Proteins (IGFBPs)	2	127	8.63 × 10^−3^	4.26 × 10^−2^	2.46 × 10^−1^	IGFBP4; IGFALS
R-HSA-166187	Mitochondrial Uncoupling	1	18	1.22 × 10^−3^	4.54 × 10^−2^	2.46 × 10^−1^	PM20D1

**Table 2:** The top 15 pathways enriched from the OA-responsive DEGs in female cartilage. Note: all pathways in the list have an FDR value less than 0.05.

Pathway Identifier	Pathway Name	#Entities Found	#Entities Total	Entities Ratio	Entities *p*-Value	Entities FDR	Submitted Entities Found
R-HSA-1474244	Extracellular matrix organization	55	330	2.24 × 10^−2^	7.42 × 10^−8^	1.06 × 10^−4^	COL18A1; SPARC; ITGAM; ELN; SERPINE1; ITGB2; TNC; HAPLN1; ADAMTS5; ADAMTS2; EFEMP1; TNN; CTSK; TNR; ITGB8; MME; ITGA4; COL25A1; PCOLCE; ASPN; VCAN; COL2A1; MMP13; OPTC; COL6A1; ADAM12; PECAM1; COL8A1; MMP19; LAMA5; COL15A1; COL13A1; HTRA1; FBLN1; LTBP2; FBLN5; ADAMTS14; SPP1; NCAM1; COL26A1; LAMB3; LUM; FN1; GDF5; COL1A1; COL3A1; CAPN12; BMP1; COL1A2; COL5A1; P4HA3; COL5A2; TLL1
R-HSA-9617828	FOXO-mediated transcription of cell cycle genes	12	27	1.83 × 10^−3^	1.50 × 10^−6^	8.48 × 10^−4^	NOTCH3; CDKN1A; CDKN1B; GADD45A; CCNG2; FOXO3; KLF4
R-HSA-9614085	FOXO-mediated transcription	25	110	7.47 × 10^−3^	1.79 × 10^−6^	8.48 × 10^−4^	IGFBP1; NOTCH3; CDKN1A; CDKN1B; GADD45A; CITED2; FOXO6; FOXO3; KLF4; FBXO32; BCL6; CCNG2; DDIT3; TXNIP; PLXNA4
R-HSA-3000178	ECM proteoglycans	20	79	5.37 × 10^−3^	4.01 × 10^−6^	1.42 × 10^−3^	LAMA5; ITGAM; SPARC; LUM; SERPINE1; FN1; TNC; HAPLN1; ASPN; COL1A1; VCAN; COL3A1; COL2A1; COL1A2; COL5A1; TNN; COL6A1; COL5A2; TNR; NCAM1
R-HSA-1650814	Collagen biosynthesis and modifying enzymes	19	76	5.16 × 10^−3^	8.23 × 10^−6^	2.34 × 10^−3^	COL18A1; COL26A1; COL15A1; COL13A1; COL25A1; PCOLCE; COL1A1; ADAMTS2; ADAMTS14; COL3A1; COL2A1; BMP1; COL1A2; COL5A1; P4HA3; COL6A1; COL5A2; COL8A1; TLL1
R-HSA-1474228	Degradation of the extracellular matrix	28	148	1.01 × 10^−3^	1.32 × 10^−5^	3.08 × 10^−3^	COL18A1; LAMA5; COL15A1; COL13A1; ELN; HTRA1; ADAMTS5; CTSK; SPP1; COL26A1; LAMB3; MME; COL25A1; FN1; COL1A1; COL3A1; MMP13; COL2A1; COL1A2; CAPN12; BMP1; COL5A1; OPTC; COL6A1; COL5A2; COL8A1; MMP19; TLL1
R-HSA-216083	Integrin cell surface interactions	20	87	5.91 × 10^−3^	1.59 × 10^−5^	3.08 × 10^−3^	COL18A1; ITGAM; COL13A1; ITGA4; LUM; ITGB2; FN1; TNC; COL1A1; COL3A1; COL2A1; COL1A2; COL5A1; COL6A1; COL5A2; SPP1; PECAM1; COL8A1; ITGB8
R-HSA-9648895	Response of EIF2AK1 (HRI) to heme deficiency	11	29	1.97 × 10^−3^	1.74 × 10^−5^	3.08 × 10^−3^	PPP1R15A; DDIT3; CEBPG; TNR; TRIB3; ATF3
R-HSA-1442490	Collagen degradation	17	69	4.69 × 10^−3^	2.88 × 10^−5^	4.22 × 10^−3^	COL18A1; COL26A1; COL15A1; COL13A1; MME; COL25A1; COL1A1; COL3A1; MMP13; COL2A1; COL1A2; COL5A1; CTSK; COL6A1; COL5A2; MMP19; COL8A1
R-HSA-6785807	Interleukin-4 and Interleukin-13 signaling	35	216	1.47 × 10^−2^	2.97 × 10^−5^	4.22 × 10^−3^	NOTCH3; LAMA5; CDKN1A; ITGAM; ITGB2; FN1; RORC; TWIST1; FOXO3; VEGFA; COL1A2; SOCS1; CCND1; BCL6; IRF4; BIRC5; IL6R; FAN1
R-HSA-8948216	Collagen chain trimerization	13	44	2.99 × 10^−3^	4.11 × 10^−5^	5.30 × 10^−3^	COL18A1; COL26A1; COL15A1; COL13A1; COL25A1; COL1A1; COL3A1; COL2A1; COL1A2; COL5A1; COL6A1; COL5A2; COL8A1
R-HSA-1474290	Collagen formation	21	104	7.06 × 10^−3^	6.24 × 10^−5^	7.36 × 10^−3^	COL18A1; COL26A1; COL15A1; COL13A1; LAMB3; COL25A1; PCOLCE; COL1A1; ADAMTS2; ADAMTS14; COL3A1; MMP13; COL2A1; BMP1; COL1A2; COL5A1; P4HA3; COL6A1; COL5A2; COL8A1; TLL1
R-HSA-8949275	RUNX3 Regulates Immune Response and Cell Migration	6	10	6.79 × 10^−4^	1.30 × 10^−4^	1.41 × 10^−2^	ITGA4; SPP1; RORC
R-HSA-2022090	Assembly of collagen fibrils and other multimeric structures	15	67	4.55 × 10^−3^	2.30 × 10^−4^	2.33 × 10^−2^	COL18A1; COL15A1; LAMB3; PCOLCE; COL1A1; COL3A1; MMP13; COL2A1; BMP1; COL1A2; COL5A1; COL6A1; COL5A2; COL8A1; TLL1
R-HSA-190861	Gap junction assembly	11	41	2.79 × 10^−3^	3.52 × 10^−4^	3.31 × 10^−2^	GJC1; PLK4; GJB2; TUBB3; TUBB4B; TUBA4A; TUBA8

**Table 3:** The top 15 pathways enriched from the OA-responsive DEGs in male cartilage. Pathways with an FDR less than 0.05 are highlighted in red.

Pathway Identifier	Pathway Name	#Entities Found	#Entities Total	Entities Ratio	Entities *p*-Value	Entities FDR	Submitted Entities Found
R-HSA-9648895	Response of EIF2AK1 (HRI) to heme deficiency	10	29	1.97 × 10^−3^	1.32 × 10^−7^	1.43 × 10^−4^	PPP1R15A; DDIT3; CEBPG; TNR; CHAC1; ATF3
R-HSA-380994	ATF4 activates genes in response to endoplasmic reticulum stress	9	34	2.31 × 10^−3^	4.71 × 10^−6^	2.56 × 10^−3^	IGFBP1; DDIT3; CEBPG; ATF3; HERPUD1
R-HSA-9031628	NGF-stimulated transcription	11	56	3.80 × 10^−3^	7.12 × 10^−6^	2.58 × 10^−3^	FOSL1; EGR1; ARC; EGR3; FOSB; FOS; TRIB1; JUNB
R-HSA-9022699	MECP2 regulates neuronal receptors and channels	8	32	2.17 × 10^−3^	2.37 × 10^−5^	5.35 × 10^−3^	GRIA2; GRIN2A; OPRK1; SLC2A3
R-HSA-381042	PERK regulates gene expression	9	42	2.85 × 10^−3^	2.46 × 10^−5^	5.35 × 10^−3^	IGFBP1; DDIT3; CEBPG; ATF3; HERPUD1
R-HSA-198725	Nuclear Events (kinase and transcription factor activation)	11	80	5.43 × 10^−3^	1.70 × 10^−4^	3.07 × 10^−2^	FOSL1; EGR1; ARC; EGR3; FOSB; FOS; TRIB1; JUNB
R-HSA-6791312	TP53 Regulates Transcription of Cell Cycle Genes	9	65	4.42 × 10^−3^	6.18 × 10^−4^	9.58 × 10^−2^	CCNA2; NOTCH3; BTG2; CDKN1A; PLK2; CDK1
R-HSA-6785807	Interleukin-4 and Interleukin-13 signaling	18	216	1.47 × 10^−2^	8.82 × 10^−4^	1.19 × 10^−1^	NOTCH3; CDKN1A; COL1A2; IRF4; ITGB2; LIF; FOS; TNFRSF1B; JUNB; VEGFA
R-HSA-6804757	Regulation of TP53 Degradation	7	43	2.92 × 10^−3^	9.81 × 10^−4^	1.19 × 10^−1^	CCNA2; USP2; UBC; CDK1; PDK1
R-HSA-69895	Transcriptional activation of cell cycle inhibitor p21	3	6	4.08 × 10^−4^	1.36 × 10^−3^	1.29 × 10^−1^	NOTCH3; CDKN1A
R-HSA-69560	Transcriptional activation of p53 responsive genes	3	6	4.08 × 10^−4^	1.36 × 10^−3^	1.29 × 10^−1^	NOTCH3; CDKN1A
R-HSA-6806003	Regulation of TP53 Expression and Degradation	7	46	3.12 × 10^−3^	1.44 × 10^−3^	1.29 × 10^−1^	CCNA2; USP2; UBC; CDK1; PDK1
R-HSA-1538133	G0 and Early G1	6	38	2.58 × 10^−3^	2.59 × 10^−3^	2.07 × 10^−1^	TOP2A; CCNA2; CDK1
R-HSA-9617828	FOXO-mediated transcription of cell cycle genes	5	27	1.83 × 10^−3^	2.99 × 10^−3^	2.07 × 10^−1^	NOTCH3; CDKN1A; KLF4
R-HSA-194313	VEGF ligand-receptor interactions	3	8	5.43 × 10^−4^	3.05 × 10^−3^	2.07 × 10^−1^	PGF; VEGFA

**Table 4:** OA-responsive DEGs that have significantly different expression fold changes between males and females, and significantly (*p* < 0.05) altered in at least one gender. DEGs significantly upregulated in response to OA are highlighted in red, and those significantly downregulated in blue.

SYMBOL	OM-HM		OF-HF		OM-HM vs. OF-HF	
	log_2_FC	*p*-Value	log_2_FC	*p*-Value	log_2_FC	*p*-Value
ADAMTS2	1.036311	4.73 × 10^−1^	4.245269	1.24 × 10^−4^	−3.208959	1.37 × 10^−2^
AKR1C2	0.716720	7.69 × 10^−1^	2.893709	2.96 × 10^−4^	−2.176989	3.56 × 10^−2^
APBB1IP	1.029711	4.80 × 10^−1^	4.594870	1.20 × 10^−4^	−3.565158	9.94 × 10^−3^
AQP1	0.781688	6.57 × 10^−1^	3.956589	2.86 × 10^−4^	−3.174901	1.43 × 10^−2^
ARMS2	−0.356654	9.08 × 10^−1^	2.345976	1.51 × 10^−2^	−2.702631	1.64 × 10^−2^
BAALC	0.980694	5.17 × 10^−1^	3.404522	5.70 × 10^−4^	−2.423828	4.66 × 10^−2^
C1QTNF1	0.619342	8.09 × 10^−1^	2.996843	2.75 × 10^−3^	−2.377501	4.77 × 10^−2^
CAVIN4	0.237081	9.28 × 10^−1^	2.976823	3.48 × 10^−3^	−2.739743	2.87 × 10^−2^
CCDC163	0.768465	7.15 × 10^−1^	−2.087867	1.36 × 10^−2^	2.856332	2.44 × 10^−3^
CDCA2	0.439866	8.37 × 10^−1^	3.382528	1.19 × 10^−3^	−2.942662	2.26 × 10^−2^
CDKL2	0.651973	7.13 × 10^−1^	−2.454556	1.72 × 10^−2^	3.106529	1.36 × 10^−2^
COL15A1	0.397324	9.18 × 10^−1^	2.601580	2.23 × 10^−3^	−2.204256	4.27 × 10^−2^
COL18A1	0.108196	9.90 × 10^−1^	2.571811	5.21 × 10^−3^	−2.463615	2.16 × 10^−2^
COL1A1	3.791243	3.33 × 10^−4^	7.250090	8.76 × 10^−7^	−3.458847	3.88 × 10^−2^
COL1A2	2.206448	2.20 × 10^−2^	5.722798	2.93 × 10^−8^	−3.516351	4.58 × 10^−3^
CYBB	1.286525	3.93 × 10^−1^	5.141565	9.37 × 10^−4^	−3.855040	3.95 × 10^−2^
DKK3	0.654166	7.59 × 10^−1^	3.287993	5.43 × 10^−4^	−2.633827	2.58 × 10^−2^
DPT	0.826854	6.55 × 10^−1^	3.052945	3.79 × 10^−4^	−2.226091	4.61 × 10^−2^
EMB	0.540007	7.61 × 10^−1^	3.865787	9.00 × 10^−4^	−3.325780	2.08 × 10^−2^
EMX2OS	−0.745100	6.41 × 10^−1^	3.025770	3.24 × 10^−2^	−3.770870	2.35 × 10^−2^
EPYC	0.082818	9.87 × 10^−1^	2.941842	4.25 × 10^−3^	−2.859024	1.52 × 10^−2^
FAN1	0.487189	7.97 × 10^−1^	−2.529764	4.07 × 10^−2^	3.016953	3.39 × 10^−2^
FBLN5	0.820198	6.62 × 10^−1^	3.185592	1.17 × 10^−3^	−2.365394	4.72 × 10^−2^
GAP43	1.465410	2.56 × 10^−1^	4.712171	6.43 × 10^−5^	−3.246761	2.64 × 10^−2^
HMGB4	0.067476	9.82 × 10^−1^	2.557579	1.19 × 10^−2^	−2.490102	4.46 × 10^−2^
HPDL	−0.057707	9.88 × 10^−1^	2.475639	1.40 × 10^−2^	−2.533346	3.20 × 10^−2^
IFI44L	0.012213	9.96 × 10^−1^	3.090819	1.51 × 10^−2^	−3.078605	3.65 × 10^−2^
IGFBP4	0.764578	6.78 × 10^−1^	3.556192	8.62 × 10^−4^	−2.791614	2.86 × 10^−2^
LINC02447	0.377013	8.91 × 10^−1^	−2.237060	2.63 × 10^−2^	2.614072	2.53 × 10^−2^
LOC100507250	0.452652	8.45 × 10^−1^	−2.527564	1.12 × 10^−2^	2.980216	1.19 × 10^−2^
LOC101929122	0.056037	9.84 × 10^−1^	2.846396	5.90 × 10^−3^	−2.790359	2.55 × 10^−2^
MIR4435-2HG	0.335817	9.45 × 10^−1^	3.246292	3.10 × 10^−4^	−2.910474	6.24 × 10^−3^
MXRA5	2.179711	4.13 × 10^−2^	5.721745	4.35 × 10^−6^	−3.542034	1.59 × 10^−2^
NEURL1B	−0.431259	8.20 × 10^−1^	2.888588	1.59 × 10^−2^	−3.319847	1.87 × 10^−2^
NGF	1.338201	3.52 × 10^−1^	5.880944	5.41 × 10^−5^	−4.542743	8.71 × 10^−3^
OGN	1.571206	1.44 × 10^−1^	3.988797	1.87 × 10^−5^	−2.417591	4.88 × 10^−2^
PALM2	0.305307	9.84 × 10^−1^	2.300331	4.37 × 10^−3^	−1.995024	4.34 × 10^−2^
PDLIM1	0.824352	6.02 × 10^−1^	5.213650	1.57 × 10^−4^	−4.389298	6.60 × 10^−3^
PECAM1	−0.523163	7.50 × 10^−1^	3.413773	3.56 × 10^−2^	−3.936936	3.52 × 10^−2^
PLAU	1.131386	4.51 × 10^−1^	4.754242	1.15 × 10^−3^	−3.622856	4.01 × 10^−2^
PLK4	0.921031	5.52 × 10^−1^	3.702119	5.00 × 10^−4^	−2.781088	3.84 × 10^−2^
RCAN1	0.462142	8.79 × 10^−1^	2.998940	8.88 × 10^−4^	−2.536799	2.38 × 10^−2^
S100A4	1.739908	8.02 × 10^−2^	4.787812	2.72 × 10^−5^	−3.047904	2.23 × 10^−2^
SERPINE1	−0.128314	9.57 × 10^−1^	3.610779	1.52 × 10^−3^	−3.739093	5.28 × 10^−3^
SERPINE2	1.067892	4.41 × 10^−1^	3.350974	1.14 × 10^−4^	−2.283082	4.46 × 10^−2^
SGIP1	−0.199640	9.58 × 10^−1^	2.341629	3.22 × 10^−2^	−2.541269	3.86 × 10^−2^
THY1	3.064184	3.65 × 10^−4^	7.253767	9.26 × 10^−7^	−4.189584	7.67 × 10^−3^
TNFAIP6	3.028402	1.71 × 10^−3^	7.264821	8.86 × 10^−6^	−4.236418	1.56 × 10^−2^
TSIX	1.327472	4.64 × 10^−1^	−3.508086	3.20 × 10^−3^	4.835558	1.38 × 10^−2^
VCAN	−0.392235	8.76 × 10^−1^	2.248223	3.39 × 10^−2^	−2.640458	3.07 × 10^−2^

**Table 5:** The top 15 pathways enriched from the DEGs that differently altered in response to OA in male and female cartilage. Pathways with an FDR less than 0.05 are highlighted in red.

Pathway Identifier	Pathway Name	#Entities Found	#Entities Total	Entities Ratio	Entities *p*-Value	Entities FDR	Submitted Entities Found
R-HSA-1474244	Extracellular matrix organization	10	330	2.24 × 10^−2^	8.68 × 10^−7^	2.38 × 10^−4^	COL1A1; COL18A1; VCAN; COL15A1; ADAMTS2; COL1A2; SERPINE1; PECAM1; FBLN5
R-HSA-1650814	Collagen biosynthesis and modifying enzymes	5	76	5.16 × 10^−3^	1.58 × 10^−5^	2.17 × 10^−3^	COL1A1; COL18A1; COL15A1; ADAMTS2; COL1A2
R-HSA-75205	Dissolution of Fibrin Clot	3	14	9.51 × 10^−4^	2.83 × 10^−5^	2.32 × 10^−3^	SERPINE2; PLAU; SERPINE1
R-HSA-8948216	Collagen chain trimerization	4	44	2.99 × 10^−3^	3.41 × 10^−5^	2.32 × 10^−3^	COL1A1; COL18A1; COL15A1; COL1A2
R-HSA-1474290	Collagen formation	5	104	7.07 × 10^−3^	6.96 × 10^−5^	3.76 × 10^−3^	COL1A1; COL18A1; COL15A1; ADAMTS2; COL1A2
R-HSA-2022090	Assembly of collagen fibrils and other multimeric structures	4	67	4.55 × 10^−3^	1.71 × 10^−4^	7.45 × 10^−3^	COL1A1; COL18A1; COL15A1; COL1A2
R-HSA-1442490	Collagen degradation	4	69	4.69 × 10^−3^	1.91 × 10^−4^	7.45 × 10^−3^	COL1A1; COL18A1; COL15A1; COL1A2
R-HSA-3000178	ECM proteoglycans	4	79	5.37 × 10^−3^	3.18 × 10^−4^	1.08 × 10^−2^	COL1A1; VCAN; COL1A2; SERPINE1
R-HSA-216083	Integrin cell surface interactions	4	87	5.91 × 10^−3^	4.57 × 10^−4^	1.37 × 10^−2^	COL1A1; COL18A1; COL1A2; PECAM1
R-HSA-430116	GP1b-IX-V activation signaling	2	12	8.15 × 10^−4^	1.14 × 10^−3^	3.08 × 10^−2^	COL1A1; COL1A2
R-HSA-76009	Platelet Aggregation (Plug Formation)	3	53	3.60 × 10^−3^	1.37 × 10^−3^	3.13 × 10^−2^	COL1A1; APBB1IP; COL1A2
R-HSA-3781865	Diseases of glycosylation	5	202	1.37 × 10^−2^	1.42 × 10^−3^	3.13 × 10^−2^	VCAN; ADAMTS2; SERPINE2; OGN; BAALC
R-HSA-2214320	Anchoring fibril formation	2	15	1.02 × 10^−3^	1.77 × 10^−3^	3.71 × 10^−2^	COL1A1; COL1A2
R-HSA-75892	Platelet Adhesion to exposed collagen	2	16	1.09 × 10^−3^	2.01 × 10^−3^	3.81 × 10^−2^	COL1A1; COL1A2
R-HSA-1474228	Degradation of the extracellular matrix	4	148	1.01 × 10^−2^	3.18 × 10^−3^	5.73 × 10^−2^	COL1A1; COL18A1; COL15A1; COL1A2

**Table 6:** The sample size and age information for each group.

Group	Sample Size	Age Range (years)	Mean Age	OA Score Range	Mean OA Score
Healthy Female	5	27–57	42 yrs	1–1	1
Healthy Male	13	18–61	34.5 yrs	1–1	1
OA Female	11	52–82	66.3 yrs	4–4	4
OA Male	9	51–71	64.9 yrs	4–4	4

## Data Availability

The data presented in this study are contained within this article and Supplementary Materials.

## Supplementary Materials

The following are available online at https://www.mdpi.com/article/10.3390/ijms22157876/s1.
